# Age-related frailty and its association with biological markers of ageing

**DOI:** 10.1186/s12916-015-0400-x

**Published:** 2015-07-13

**Authors:** Arnold Mitnitski, Joanna Collerton, Carmen Martin-Ruiz, Carol Jagger, Thomas von Zglinicki, Kenneth Rockwood, Thomas B. L. Kirkwood

**Affiliations:** Department of Medicine, Dalhousie University, Halifax, NS B3H 2E1 Canada; Institute of Health and Society and Newcastle University Institute for Ageing, Newcastle upon Tyne, NE4 5PL UK; Institute of Neuroscience and Newcastle University Institute for Ageing, Newcastle upon Tyne, NE4 5PL UK; Institute for Cell and Molecular Biosciences and Newcastle University Institute for Ageing, Newcastle upon Tyne, NE4 5PL UK; Division of Geriatric Medicine, Dalhousie University, Halifax, NS B3H 2E1 Canada

**Keywords:** Ageing, Biomarkers, Cellular ageing, Deficit accumulation, Frailty, Frailty index, Frailty phenotype, Immunosenescence, Inflammation, Newcastle 85+ study

## Abstract

**Background:**

The relationship between age-related frailty and the underlying processes that drive changes in health is currently unclear. Considered individually, most blood biomarkers show only weak relationships with frailty and ageing. Here, we examined whether a biomarker-based frailty index (FI-B) allowed examination of their collective effect in predicting mortality compared with individual biomarkers, a clinical deficits frailty index (FI-CD), and the Fried frailty phenotype.

**Methods:**

We analyzed baseline data and up to 7-year mortality in the Newcastle 85+ Study (n = 845; mean age 85.5). The FI-B combined 40 biomarkers of cellular ageing, inflammation, haematology, and immunosenescence. The Kaplan-Meier estimator was used to stratify participants into FI-B risk strata. Stability of the risk estimates for the FI-B was assessed using iterative, random subsampling of the 40 FI-B items. Predictive validity was tested using Cox proportional hazards analysis and discriminative ability by the area under receiver operating characteristic (ROC) curves.

**Results:**

The mean FI-B was 0.35 (SD, 0.08), higher than the mean FI-CD (0.22; SD, 0.12); no participant had an FI-B score <0.12. Higher values of each FI were associated with higher mortality risk. In a sex-adjusted model, each one percent increase in the FI-B increased the hazard ratio by 5.4 % (HR, 1.05; CI, 1.04–1.06). The FI-B was more powerful for mortality prediction than any individual biomarker and was robust to biomarker substitution. The ROC analysis showed moderate discriminative ability for 7-year mortality (AUC for FI-CD = 0.71 and AUC for FI-B = 0.66). No individual biomarker’s AUC exceeded 0.61. The AUC for combined FI-CD/FI-B was 0.75.

**Conclusions:**

Many biological processes are implicated in ageing. The systemic effects of these processes can be elucidated using the frailty index approach, which showed here that subclinical deficits increased the risk of death. In the future, blood biomarkers may indicate the nature of the underlying causal deficits leading to age-related frailty, thereby helping to expose targets for early preventative interventions.

**Electronic supplementary material:**

The online version of this article (doi:10.1186/s12916-015-0400-x) contains supplementary material, which is available to authorized users.

## Background

The ageing process is characterized by declining functional capacity and increasing vulnerability to disease, disability, and death. This is driven by the gradual, lifelong accumulation of molecular and cellular defects [1]. Individuals of a given chronological age vary, however, in how far these processes have advanced. Some enter a state of increased risk, compared with others of the same age, known as frailty [2]. Connecting the clinical state of age-related frailty with underlying biological age, as measured by biomarkers, has long been an elusive goal.

Frailty can be evaluated, and health status quantified, by the number of health deficits that individuals accumulate. Specifically, the frailty index (FI) is the ratio of the deficits present in a person to the total number of potential deficits evaluated [3]. The FI has been used in many epidemiological and clinical studies to grade the degree of risk of several adverse outcomes, including mortality, health service use, hospital-acquired complications, worsening health, and loss of independence [4–9], as well as conditions such as cognitive impairment [10], heart disease [11], osteoporosis [12], intellectual disability [13], and systemic sclerosis [14]. In most studies, the FIs have been made up of clinically-assessed health deficits [9].

In a comprehensive study of biomarkers of cellular ageing, inflammation, and immunosenescence, Collerton et al. [15] identified several biomarkers associated with frailty (assessed both by the FI and the Fried frailty phenotype [16]), where each biomarker was considered separately from the others. Although such an analysis can identify important specific effects, it is known that complex interactions between ageing mechanisms and biomarkers are likely [17, 18]. Within the present work, we significantly extend our previous analyses by combining different biomarker measures into a FI, which may make it possible to discern a systemic effect that might be masked when the variables are used in isolation [19].

In principle, a FI of clinical health deficits should reflect the accumulation of damage at the level of the organism due to impairment of macroscopic and microscopic repair processes [20]. We were therefore interested to know whether combining biomarkers of deficits at the levels of tissue (anaemia, inflammatory markers) and cells (e.g., DNA damage, telomere length) would add to the ability of a FI based on clinical deficits (Clinical Deficits Frailty Index; FI-CD) to predict adverse outcomes.

Our objectives were 1) to develop a FI based on biomarkers (Biomarker Frailty Index; FI-B); 2) to investigate the robustness of the FI-B in relation to the specific biomarkers included; and 3) to test its predictive value in relation to death, compared to other frailty measures (FI-CD, Fried phenotype) and individual biomarkers.

## Methods

### Participants

As detailed elsewhere, the Newcastle 85+ Study is a population-based cohort study of people born in 1921, aged 85 at recruitment, living in Newcastle upon Tyne and North Tyneside (North East England); the cohort was socio-demographically representative of the local population and of England and Wales [21–23]. The research complied with the requirements of the Declaration of Helsinki. Ethical approval was obtained from the Newcastle and North Tyneside 1 Research Ethics Committee (reference number 06/Q0905/2). Written informed consent was obtained from participants and where people lacked capacity to consent, for example because of dementia, a formal written opinion was sought from a relative or carer. Participants could decline any or all elements of the protocol.

### Data sources

A multidimensional health assessment was carried out in the participant’s usual residence by a research nurse. Data on pre-existing diseases and prescribed medications were obtained from general practice medical records.

### Blood-based biomarkers

Biomarkers were measured from a blood sample drawn between 7 am and 10:30 am following an 8 h overnight fast and delivered to the laboratory for initial processing within 1 hour of draw. The 40 biomarkers investigated included inflammatory, haematological, immunological, cell senescence, genetic, and epigenetic markers, and were analyzed according to either previously reported methods [24] or those described in Additional file 1: Material and Methods.

### Recoding (dichotomizing) biomarkers as deficits

Each biomarker was measured on a continuous scale and values were dichotomized into ‘deficit’ versus ‘no deficit’ using empirical cut points, chosen to achieve the best separation of survival curves between people with and without the deficit by minimizing the *P* value of the log rank test (Table 1, Additional file 1: Figure S1).

**Table 1:** Individual biomarkers used to compose the frailty indices (FI-B). The cut off points were defined to achieve the best separation of survival curves between people with and without the deficit and minimizing the *P* value of the log rank test

Biomarker	Cut off point	Direction of risk	Number of participants		*P* value**
			At lower risk	At higher risk	
Inflammation*					
Cytomegalovirus serology (IgG)	Positive		108	641	0.327
High sensitivity C-reactive protein (mg/L)	>25	High	737	37	**<0.001**
IL-6, basal (pg/mL)	>15	High	27	676	0.399
IL-6, post-stimulation (pg/mL)	>1100	High	176	527	0.92
TNF-alpha, basal (pg/mL)	<95	Low	55	637	0.646
TNF-alpha, post-stimulation (pg/mL)	>80	High	60	643	0.116
Leptin (ng/mL)	<40	Low	214	495	**0.023**
Adiponectin (μg/mL)	>20	High	657	83	**0.012**
Homocysteine (μmol/L)	>30	High	422	35	**0.006**
Albumin (g/L)	<40	Low	365	397	**<0.001**
Haematological					
Haemoglobin (g/dL)	<11	Low	676	76	**<0.001**
Platelets (×10^9^/L)	<170	Low	675	73	**0.003**
White blood cells (×10^9^/L)	>7	High	470	274	**0.035**
Neutrophils (×10^9^/L)	>7	High	715	29	**0.007**
Lymphocytes (×10^9^/L)	<1.5	Low	530	214	**0.028**
Monocytes (×10^9^/L)	>0.75	High	639	105	0.14
Basophils (×10^9^/L)	<0.06	Low	156	588	0.156
Eosinophils (×10^9^/L)	>0.5	High	688	56	0.15
Immunosenescence					
CD4 T cells (% T cells)	<44	Low	345	367	**0.029**
CD8 T cells (% T cells)	>35	High	579	128	**0.015**
CD8 TEMRA T cells (% CD8 T cells)	>0.6	High	672	36	**0.014**
Senescent Memory CD4 T cells (% Memory CD4 T cells)	>70	High	618	93	0.079
Memory CD4 T cells (% CD4 T cells)	>35	High	672	43	0.057
CD4/CD8 T cell ratio	<0.6	Low	662	31	**0.005**
Memory/naïve CD4 T cell ratio	>3.3	High	692	20	**0.034**
Memory/naïve CD8 T cell ratio	>15	High	677	20	0.352
Memory/naïve B cell ratio	>2.5	High	652	36	**0.026**
Cellular ageing/Oxidative stress					
Telomere length (bp)	<3000	Low	634	66	0.118
DNA repair (%)	<20	Low	589	156	0.078
DNA damage/DNA repair ratio	>6.5	High	688	44	0.063
TGF beta, transforming growth factor beta (ng/mL)	<20	Low	102	644	**0.003**
IGFBP1, insulin-like growth factor-binding protein 1 (ng/mL)	>150	High	574	162	**0.001**
IGFBP3, insulin-like growth factor-binding protein 3 (ng/mL)	<800	Low	92	652	0.357
iPF2alpha-III (LC/MS/MS) (ng/mL)	<4.5	Low	85	629	**0.053**
iPF2alpha-VI (LC/MS/MS) (ng/mL)	<4	Low	423	291	0.1
iPF2alpha-III (AutoDELFIA) (ng/mL)	<0.6	Low	512	103	0.258
Genetic/Epigenetic					
Mitochondrial DNA haplogroup	X, I, heteroplasmic		675	30	**0.031**
APOE genotype	E4		454	167	**0.024**
CPG island DNA methylation (%)	>7	High	28	451	0.67
Line1 DNA methylation, surrogate for genome-wide DNA methylation (%)	>80	High	73	197	0.63

*The recorded cytokine levels may be perceived as higher than usual. Because we wanted to measure basal and post-stimulated cytokines, we measured cytokines in non-coagulated LiHe blood, using blood supernatants, not serum. Furthermore, we used an electrochemiluminescent method (MSD technology) that in our hands is much more sensitive than standard ELISA assays. Both factors are likely to explain our observed cytokine levels

**Statistically significant P-values (≤0.05) are shown in bold

### Frailty measures

The FI-B was constructed by combining results for up to 40 biomarkers. For each dichotomized biomarker, a standard procedure was followed such that zero equals the absence of the deficit and 1 equals its presence. For any individual participant, the number of deficits was summed and divided by the number of potential deficits evaluated. In consequence, an individual with no deficits would have an FI = 0, and someone with every deficit present would have an FI = 1, although previous work has shown that empirically the ceiling for FIs is generally observed at a score of 0.7 or less [4]. The FIs were calculated only if more than 80 % of the component variables were available for a given individual. The FI-B was calculated in 777 participants. For illustrative purposes, we considered four FI-B strata (low, low-to-intermediate, intermediate-to-high, and highest risk of mortality) defined using empirical cut-points of 0.25, 0.38, and 0.50, respectively, based on maximum separation of mortality curves.

The FI-CD had been calculated earlier from 40 clinical variables in 811 participants [15]. In the same previous work, the Fried frailty phenotype had also been derived in 552 participants (the chief reason why the sample for the Fried frailty phenotype was substantially lower that for the FIs was the exclusion, as per the stipulated Fried methodology, of participants with conditions which might cause them to score as frail as a result of that condition alone; in brief, reasons for exclusion were stroke, Parkinson’s disease, mini-mental state examination score of less than 18, or taking drugs for dementia, Parkinson’s disease, or depression).

### Data analysis

Kaplan-Meier survival and Cox proportional hazard models were used to estimate the probability of survival, in which FI values were converted to 0–100 integers by rounding them after multiplying them by 100, giving equal percent increments for modelling. To evaluate the robustness of the separation of the FI-B strata, we randomly selected up to 30 out of 40 biomarkers/deficits and repeated the Kaplan-Meier survival analysis 1,000 times. We also compared different versions of the FI-B to address whether effects were cumulative or driven by just a few biomarkers.

The hazard ratios (HR) of the FIs (FI-B and FI-CD) were adjusted for sex, and considered separately and together. Receiver operating characteristic (ROC) analysis was used to assess the discriminative ability of the FIs, separately and in combination, as well as the Fried frailty phenotype and individual biomarkers, in relation to mortality. The confidence intervals for the ROC were calculated using bootstrapping, with 1,000 replications. Data analysis was conducted using SPSS Version 21 (IBM SPSS.) The statistical significance level was set at *P* <0.05.

## Results

### Biomarker-based frailty index (FI-B)

The mean age of the sample of 777 people in whom the FI-B could be calculated was 85.5 years (SD, 0.4). Most were women (60.9 %). The FI-B and FI-CD samples did not differ significantly in sex, years of education, percent smokers, body mass index, or cognition (Additional file 1: Table S2).

**Fig. 1:**
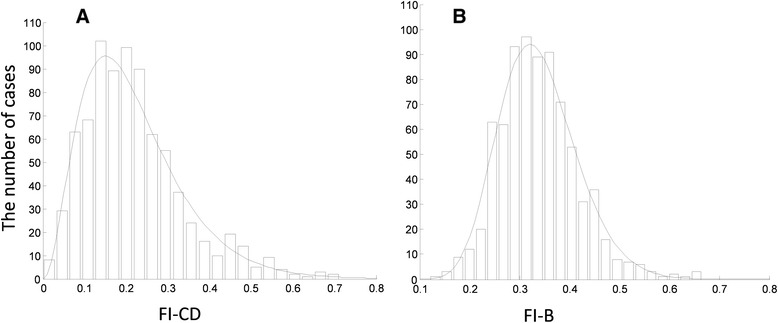
Histograms of the **a** Clinical Deficit Frailty Index (FI-CD) and **b** Biomarker Frailty Index (FI-B), and the best fit gamma density functions (solid lines) with the parameters of shape and scale 18.77 and 0.02 for FI-CD and 3.24 and 0.07 for FI-B, respectively

The FI-B showed a slightly skewed distribution, fitted by the gamma density function with shape and scale parameters of 18.77 and 0.02, respectively (Fig. 1, Panel b). The 5th centile of the FI-B was 0.24, and the 95th and 99th centiles were 0.48 and 0.60, respectively. The histogram of the FI-CD was more skewed with a longer right tail (Fig. 1, Panel a); the gamma fit shape and scale parameters were 3.24 and 0.07, respectively. The 5th centile of the FI-CD was 0.06, and the 95th and 99th centiles were 0.46 and 0.59, respectively. The mean FI-B was 0.35 (SD, 0.08), higher than the mean FI-CD of 0.22 (SD, 0.12). Individual values of the FI-B were weakly correlated with the FI-CD (r = 0.16, *P* <0.001). On average, the clinically fittest people (those with 0 or 1 clinical deficits, i.e., FI-CD = 0 or 0.02) had an average FI-B value of 0.33 (CI, 0.32–0.34), compared with an average FI-B value of 0.39 (CI, 0.36–0.41) for the clinically frailest people (FI-CD >0.5).

Of the 40 biomarkers used to compose the FI-B, 21 showed significant differences (*P* <0.05) in mortality between people with and without each deficit (Table 1). Of those 21 biomarkers, nine demonstrated a high separation of survival curves, with log rank *P* <0.001.

**Fig. 2:**
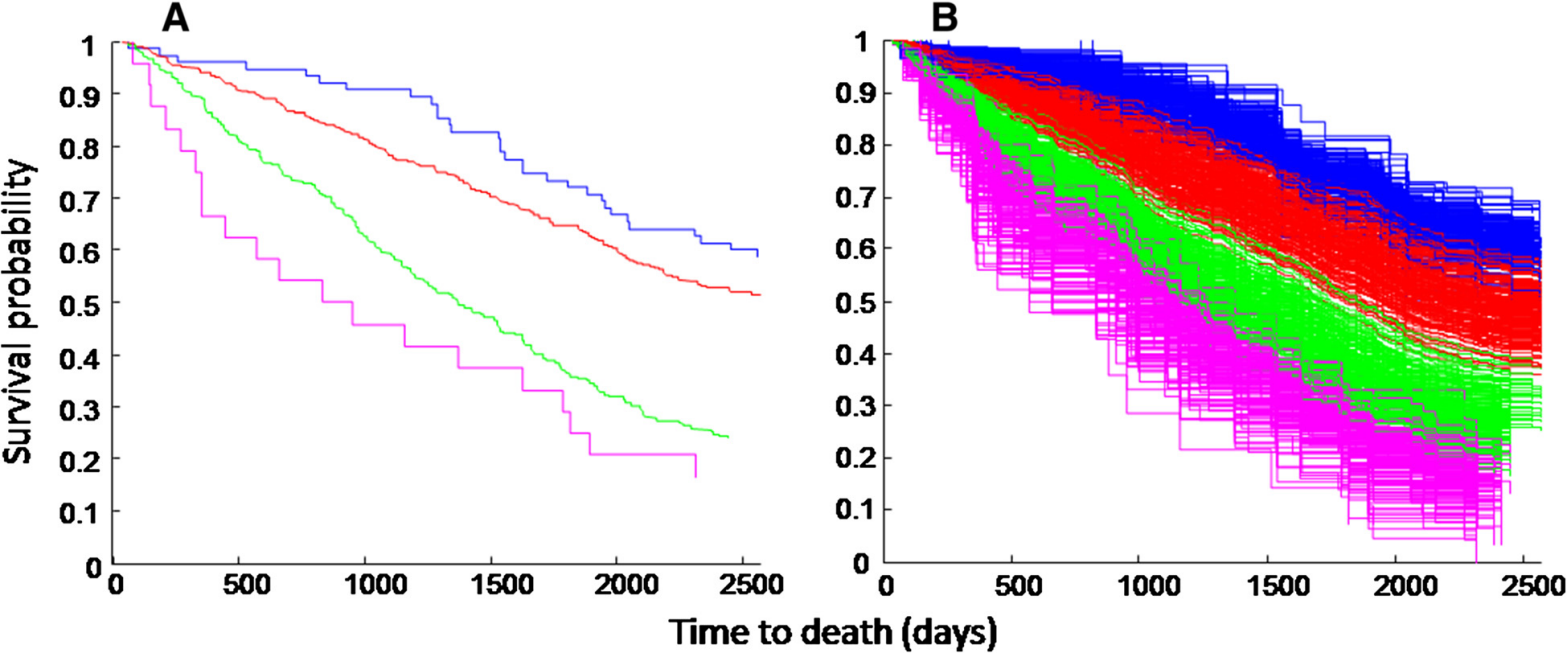
**a** Kaplan-Meier survival curves of the FI-B for the four risk strata defined by the following cut points: blue <0.25 (low risk, n = 31), red 0.25–0.38 (low-intermediate risk, n = 217), green 0.38–0.49 (intermediate-high risk, n = 154), pink ≥0.50 (highest risk, n = 32). **b** Kaplan-Meier survival curves of the FI-B calculated from 30 biomarkers randomly chosen from the total 40 and stratifying them in four groups with the same cut points as in A. The sampling was repeated 300 times

**Fig. 3:**
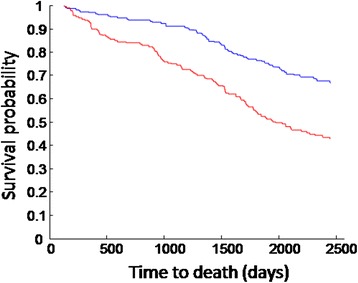
Survival of people who were not clinically frail (FI-CD < lower quartile) differs by the value of their FI-B: red for those with higher than median FI-B and blue for those with lower than median FI-B

The FI-B was strongly associated with 7-year mortality. In the Cox proportional hazards model adjusted for sex (female sex is protective; HR, 0.73; CI, 0.60–0.88), each one percent increase in FI-B was associated with 5.4 % increase in the hazards ratio (HR, 1.05; CI, 1.04–1.07). Likewise, the Kaplan-Meier survival curves showed the effect of increasing frailty across the four FI-B strata (Fig. 2a). This pattern was robust: random sub-samplings of 30 biomarkers out of the 40 available also showed good separation between the four strata, with only little overlap between neighbouring groups (Fig. 2b). With decreasing numbers of biomarkers included, the overlap between the groups greatly increased (Additional file 1: Figure S2). Notably, amongst those who clinically were not frail (FI-CD scores in the lowest quartile) having an FI-B higher than median (0.33) was associated with much higher mortality (Fig. 3).

### Comparing different versions of the FI-B

We investigated three additional versions of the FI-B. The first (FI-B-9) was calculated from the nine biomarkers that were individually highly significantly (*P* <0.001) associated with mortality in univariate analysis. Likewise, the FI-B-21 was calculated from all 21 significant (*P* <0.05) biomarkers and the third group (FI-B-NS-19) from the remaining 19 biomarkers that individually had shown no significant association with mortality (*P* >0.05). In the Cox regression analysis, each FI-B (comprised from 9, 21, and 19 items) was significantly associated with mortality. The strongest association was seen with the FI-B-9 (HR, 1.03; CI, 1.03–1.04) and the FI-B-21 (HR, 1.03; CI, 1.03–1.04) followed by the FI-B-NS-19 (HR, 1.01; CI, 1.01–1.02). These relationships did not change after adjustment for sex and the FI-CD.

### Performance of the various frailty measures

The ROC analysis showed moderate discriminative ability in relation to 7-year mortality for the different frailty measures (Table 2). The lowest area under the curve (AUC) value for the FI-B versions was for the FI-B-NS-19 (AUC = 0.60). The FI-CD had the highest predictive accuracy, with AUC = 0.71. The AUC further increased when both the FI-B and FI-CD were entered into the model (AUC = 0.75), and slightly but insignificantly increased after adjusting for sex (AUC = 0.77). Interestingly, the biomarkers considered individually had rather poor discriminative ability: the AUC did not exceed 0.61 (CI, 0.52–0.64; albumin).

**Table 2:** The area under the receiving operating characteristic (AUC) with 95 % confidence intervals for different versions of the frailty index (FI)

	AUC	95 % CI
FI-B	0.66	0.62–0.69
FI-B-21	0.68	0.62–0.73
FI-B-9	0.65	0.61–0.69
FI-B-NS	0.60	0.52–0.64
FI-CD	0.71	0.67–0.74
FI-CD + FI-B	0.75	0.71–0.78
FI-CD + FI-B + sex	0.77	0.74–0.81

Not included in Table 2 is the AUC value obtained for the Fried frailty phenotype, which was 0.58 (95 % CI, 0.53–0.63), slightly (but not significantly) lower than the lowest AUC value for the FI-B versions (FI-B-NS-19; AUC = 0.60). Since the exclusions that were necessary to produce the data for the Fried frailty phenotype might have resulted in a generally healthier subsample, which could potentially have biased the comparison, the ROC analyses for the various frailty indices were also performed using only this subsample (Additional file 1: Table S3). When this was done the predictive accuracy of the various FIs was reduced but they retained higher AUC values than for the Fried frailty phenotype. Adding the Fried phenotype to FI-CD and FI-B did not change the AUC.

## Discussion

### Overview of the findings

In this population-based sample of very old people, combining even ‘small-effect’ biomarkers in a FI resulted in a strong association with mortality. A FI produced from 40 biomarkers (and variants of it, based on statistical criteria) stratified people into four mortality risk groups. The FI-B was robust to inclusion/exclusion of individual biomarkers: any 30 randomly selected biomarkers demonstrated the same patterns of relationship to death. Such patterns became degraded, with greater overlap between the neighbouring groups, with decreasing numbers of biomarkers included (Additional file 1: Figure S2). The combination of the FI-B and a clinical FI, FI-CD, significantly improved mortality prediction compared to either index alone.

### Comparing the FI-B and FI-CD

Although the FI-B and FI-CD were significantly but weakly correlated, they showed different distributions. On average, the clinically fittest people (those with 0 or 1 clinical deficits, i.e., FI-CD, 0 or 0.02) had an average FI-B value of 0.33 (CI, 0.32–0.34). The 95th and 99th centiles for both frailty indices were very similar (0.46 for FI-CD and 0.48 for FI-B for the former; 0.59 for FI-CD and 0.60 for the latter), whereas the 5th centiles were different (0.06 for FI-CD vs. 0.24 for FI-B). This suggests that, at low FI-CD values, the FI-B is a more sensitive measure of health, i.e., it indicates important subclinical deficit accumulation (Fig. 3) whereas the frailest group is frail by either measure. The observation that FI-B is a stronger predictor of mortality in the group who were not clinically frail points to a question: do people with low FI-CD and high FI-B become clinically frail before death? To answer this will require follow-up data on the people who were non-frail in an earlier phase of assessment. Even so, the FI-B was not as closely associated with mortality as was the FI-CD, suggesting that the added value of biomarkers is highest in people without detectable clinical deficits. Note too that in both distributions, the right tail shows that 99.5 % of people had an FI value <0.7 in either the FI-B or the FI-CD. The reproducibility of this observation [7, 25–28] is consistent with a system property that has yet to be elucidated.

### Combining the FIs

Combining the FI-B and FI-CD resulted in an increase in their discriminative ability (AUC = 0.76; compared to 0.68 for FI-B and 0.71 for FI-CD, separately). This indicates that the indices are complementary to each other. At this age, the clinically fittest people as measured by the FI-CD, still have an important number of biomarker abnormalities, as reflected by their high mean FI-B scores of 0.32, well above the usual levels in a FI-CD of 0.20–0.25 that would define a person as “frail” using a dichotomous definition. Even so, changes that are subclinical alone appear to be less related to mortality; to what extent they predict adverse outcomes other than mortality will be of interest. Interestingly, the AUC for the combination of FI-CD with the FI-B-NS-19 and adjusted for sex was 0.75, suggesting further that the additive effect of items is important, even for those that individually might not be statistically significant.

### IL-6 and TNFα data

It was interesting that neither basal nor stimulated non-coagulated cytokine production were associated with 7-year survival. This is in accordance with our earlier report of absence of an association between these inflammatory markers and 18-month survival [24], although basal IL-6 was earlier associated with frailty (both Fried frailty and the FI-CD) [15]. We are aware that serum IL-6 and TNFα levels have been associated with survival in other studies. There are a number of speculations possible regarding the comparative predictive power of cell-based versus serum-based measures, but we believe these would be premature at this stage.

### Strengths and limitations

Our data must be interpreted with caution. Often, abnormalities are expressed only in one direction (i.e., only values above (or below) a reference level are considered ‘abnormal’) [15]. Our approach was strictly empirical: for each dichotomized biomarker variable, we tested whether the highest or lowest scores were associated with mortality. The potential for increased risk at both ends of the normal range has been observed for several characteristics [29–31]. Even so, we did not find evidence of a U-shaped risk of mortality for any of these biomarkers (Table 1). Here, whichever direction was associated with increased mortality was scored as a deficit for that item (Table 1 and Additional file 1: Figure S1), as has been done for all versions of the FI. This method of identifying cut-points is likely to be related to mortality within studies, so that it is not clear how generalizable it is to other populations. This needs to be evaluated. On the other hand, percentile-based cut-points, e.g., tertiles [32] or twentieth percentile rule [33], also depend on the population sample considered in those studies. Often, such cut-points are not well established, so that we were interested to note that our algorithm-identified cut-point for telomere length was virtually identical to what others have suggested [34–36]. The fact that we used mortality data to establish cut-points in order to dichotomise the biomarker values, and later used the resulting aggregate FIs to predict mortality, could also be considered a limitation. However, this was necessary given the lack of independent data, and any distortion is likely to have been small. As future data accrue, it should become possible for cut-point determination to be done on one population and prediction on another. Participants were all over the age of 85 years, which can limit generalizability. On the other hand, there is a high prevalence of clinical frailty in this under-investigated group. Furthermore, at this age, for a modest sample size – and budget – many events are likely to accrue.

Our study has several strengths. An important aspect of this study is the population-based nature of the sample [15]. In addition to the sample size and the range of biomarkers considered, it is the first study in which the FI approach has been applied to biomarkers. The FI-B shared many properties with the FI-CD, although it adds important value to the latter in identifying people at increased risk of death. The availability of a large number of biomarkers in this study allowed us to make a direct comparison of the different frailty measures, and also to assess the robustness of the FI-B to its composition of individual biomarkers. We have indicated how the algorithm for biomarker cut-points can be defined, which, coupled with earlier publications [9, 37, 38] on a standard procedure for creating a FI, allows for replication of the results by others.

### Frailty and ageing: conceptualisation

Although, as generally agreed, frailty is an age-related state of vulnerability to stressors, there are different ways to operationalize it. Frailty as defined by a clinical phenotype exposes the consequences of functional deterioration and indicates proximate factors that render the frail individual more likely to die. By contrast, if frailty can be related to biological markers of aging, these relationships should come closer to identifying the underlying factors that give rise to these functional declines. Within the frailty phenotype approach, the focus has been on clinical features of frailty; while it has been acknowledged that these need ultimately to be understood in terms of biological mechanisms, the connection has not yet been established. On the other hand, the FI approach has been chiefly one based on pragmatic scoring of multiple clinical deficits, to which subclinical ones can be added [39]. Thus, its potential utility is that the FI-B could be used as a pre-clinical measure to identify at-risk individuals before changes are apparent on clinical examination. The FI-B might also help to inform the mechanistic connections between frailty and the underlying biology of intrinsic aging. This framework also allows a practical means of addressing a fundamental fact of old age, which is, as pointed out in a recent *Nature* commentary [40], that its problems come as a package.

## Conclusions

Combining biomarkers in a FI, even those not individually associated with mortality, improves mortality prediction much beyond that of individual biomarkers. Additionally, combining a FI based on biomarkers with one based on clinically detectable deficits further improves the performance in identifying vulnerable people who are at risk of death. Finally, that so many items can contribute to mortality prediction reflects the systemic nature of ageing and mortality – health status depends on the systemic effect of multiple characteristics, of which those located within the individual can be summarized in a FI.

## Additional file

Additional file 1: Figure S1. Kaplan-Meier survival curves of the individual biomarkers split by two groups using the cut points chosen to achieve the best separation of survival curves between people with and without the deficit and minimizing the p-value of the log rank test (Table 1). Red lines show survival curves for the group “at higher risk” and the blue lines are for “at lower risk” individuals.

## Abbreviations

AUC: Area under the curve
FI: Frailty index
FI-B: Frailty index based on biomarkers
FI-CD: Frailty index based on clinical deficits
HR: Hazard ratios
ROC: Receiver Operating Characteristic
