# Supplementary material for: Age-related frailty and its association with biological markers of ageing
Source: BMC Med. 2015 Jul 13;13:161. doi: 10.1186/s12916-015-0400-x (PMC4499935; doi:10.1186/s12916-015-0400-x)
Supplement: Additional file 1: Figure S1. — Kaplan-Meier survival curves of the individual biomarkers split by two groups using the cut points chosen to achieve the best separation of survival curves between people with and without the deficit and minimizing the p-value of the log rank test (Table 1). Red lines show survival curves for the group “at higher risk” and the blue lines are for “at lower risk” individuals. [file 12916_2015_400_MOESM1_ESM.docx]

**Supplementary material and methods**

Additional biomarkers not included in previous publications were Leptin, Adiponectin, Transforming growth factor beta (TGF-beta) and Insulin-like growth factor-binding proteins 1 and 3 (IGFBP1 and IGFBP3). For all of these biomarkers, their corresponding Sandwich ELISA R&D DuoSet Development System protocols (R&D Systems, Abingdon, UK) were tested and suitably modified to be transferred onto a 384-well plate platform with the use of an EpMotion 5075 Liquid Handling robot. All colorimetric measurements were performed at 450nm with wavelength correction at 550nm on a FLUOstar Omega microplate reader (BMG Labtech, Aylesbury, UK) applying a four-parameter logistic (4-PL) curve fit to the respective standard curves. Table S1 describes specific details of the protocols.

For details of previously reported biomarkers not included in ^24^, see the following references: ^S1^ for mitochondrial haplogroups, ^S2^ for DNA methylation, and ^S3^ for APOE genotype.

Supplementary references

S1. Collerton J, Ashok D, Martin-Ruiz C, et al. Frailty and mortality are not influenced

by mitochondrial DNA haplotypes in the very old. *Neurobiol Aging*. 2013; **34**:

2889 e1-4.

S2. Collerton J, Gautrey H, Otterdijk S, et al. Acquisition of aberrant DNA

methylation is associated with frailty in the very old: findings from the

Newcastle 85+ Study. *Biogerontology*. 2014; **15**: 317-28.

S3. Deelen, J; Beekman, M; Uh, H-W; Broer, L, et al., Genome-wide association meta-

analysis of human longevity identifies a novel locus conferring survival beyond

90 years of age. *Hum. Mol. Genet*. 2014; **23**: 4420-32.

Supplementary Figures and Table legends

Figure S1. Kaplan-Meier survival curves for dichotomized biomarkers. Kaplan-Meier survival curves of the biomarkers split by the two groups using the cut points (Table 1). Red lines show survival curves for the individuals who are “at higher risk” and the blue lines are for “at lower risk” individuals.

Figure S2. Kaplan-Meier survival curves of the FI-B calculated from biomarkers (the number is indicated on the top of each panel) randomly chosen from the total 40 and stratifying them in four groups with the same cut points as in Figure 3. The sampling is repeated 100 times.

Table S1. Specific details of the protocols for additional biomarkers.

Table S2. Key socio-demographic and health parameters reported for samples with FI-CD and FI-B. Comparison of the FI-CD sample with FI-B sample.

Table S3. The area under the receiving operating characteristic (AUC) with 95% confidence intervals for different versions of the FI and Fried phenotype in the subsample of participants for whom the Fried phenotype was available (n=552).

Figure S1. Kaplan-Meier survival curves of the individual biomarkers split by two groups using the cut points chosen to achieve the best separation of survival curves between people with and without the deficit and minimizing the p-value of the log rank test (Table 1). Red lines show survival curves for the group “at higher risk” and the blue lines are for “at lower risk” individuals.

INFLAMMATION

Figure S1 continued

HAEMATOLOGICAL

Figure S1 continued

IMMUNOSENESCENCE

Figure S1 continued

CELLULAR AGEING/ OXIDATIVE STRESS

Figure S1 continued

GENETIC/EPIGENETIC

Figure S2. Kaplan-Meier survival curves of the FI-B calculated from biomarkers (the number is indicated on the top of each panel) randomly chosen from the total 40 and stratifying them in four groups with the same cut points as in Figure 2. The sampling is repeated 100 times.


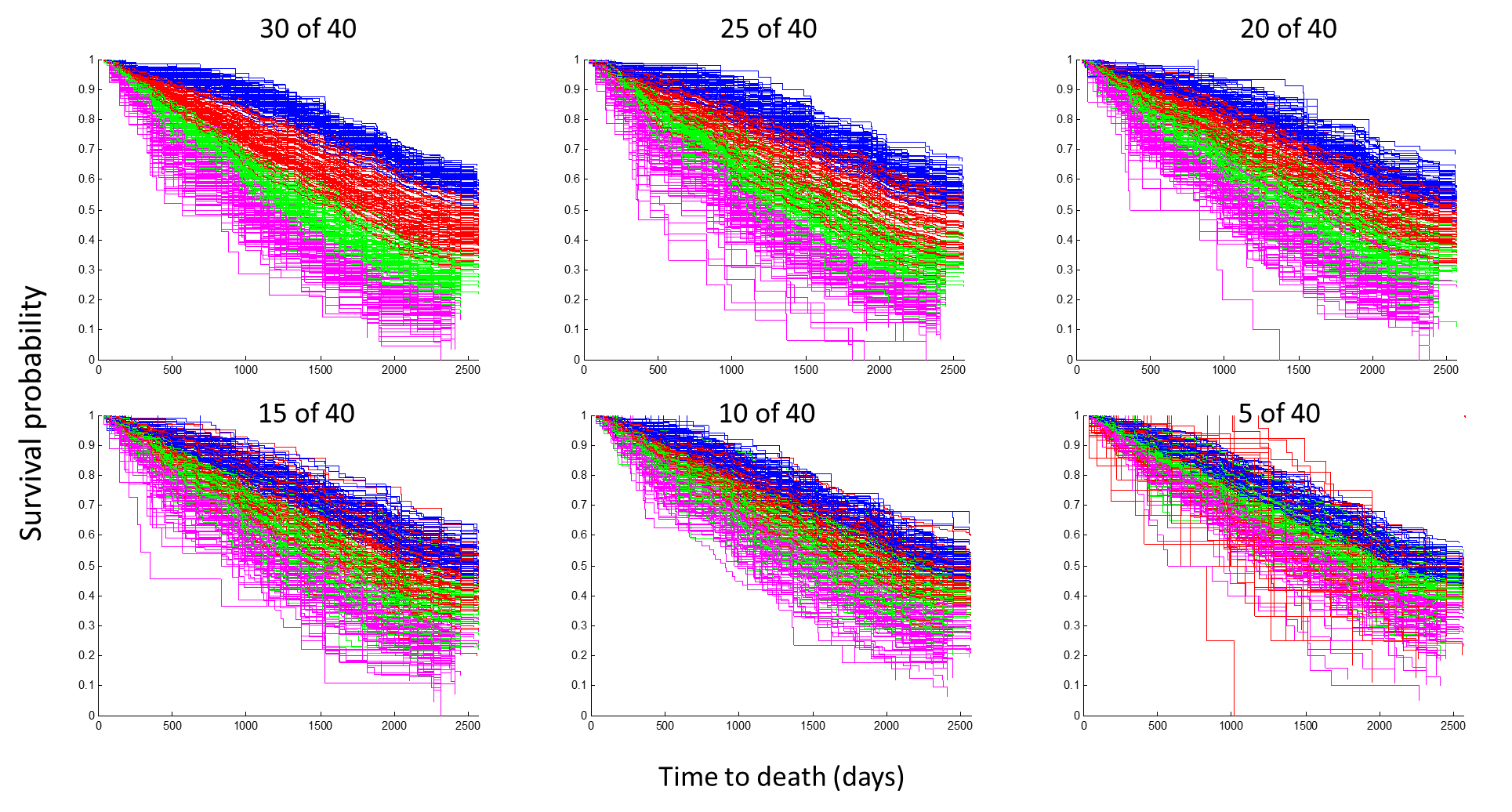


Table S1. Specific details of the protocols for additional biomarkers.

|  | Biomarker | | | | |
| --- | --- | --- | --- | --- | --- |
|  | Leptin | Adiponectin | TGF-beta | IGFBP1 | IGFBP3 |
| R&D Duoset | DY398 | DY1065 | DY240 | DY871 | DY675 |
| Sample | Serum | Serum | Serum | Serum | Serum |
| Dilution | 1:40 | 1:6000 | 1:20 | 1:100 | 1:200 |
| Dilution Conditions | Reagent Diluent DY995 including 1%BSA | Reagent Diluent DY995 including 1%BSA | Activation with 1N HCl | Reagent Diluent DY004 including 5% Tween 20 | Reagent Diluent DY004 including 5% Tween 20 and 2% Normal Goat Serum |
|  |  |  | Neutralisation with 1.2N NaOH/0.5M HEPES |  |  |
|  |  |  | Reagent Diluent DY997 including 0.05% Tween 20 |  |  |
| Capture Antibody concentration | 1.33ug/ml | 1.0ug/ml | 2.0 µg/ml | 4.0ug/ml | 4.0ug/ml |
| Detection Antibody concentration | 4.16ng/ml | 1.0ug/ml | 300 ng/ml | 400ng/ml | 200ng/ml |
| Standard Curve Range | 0.5ng/ml to  156.25pg/ml | 0.6ng/ml to  187.5pg/ml | 2ng/ml to  62.5 pg/ml | 2ng/ml to  62.5pg/ml | 8ng/ml to  250pg/ml |
| Inter-assay CV | 4.21% | 7.48% | 3.32% | 4.89% | 4.43% |
| Intra-assay CV | 2.72% | 6.06% | 6.03% | 5.42% | 4.52% |

Table S2. Key socio-demographic and health parameters –reported for samples with FI-CD and FI-B. Comparison of the FI-CD sample with FI-B sample.^a^

Table S2. Key socio-demographic and health parameters reported for samples with FI-CD and FI-B. Comparison of the FI-CD sample with FI-B sample.

|  | Sample with FI-CD | Sample with FI-B | p value^b^ |
| --- | --- | --- | --- |
|  | n=811 | n=777 |  |
| **Female** | 61·6 (500) | 60·9 (473) | 0·822 |
| **Years of Education** |  |  |  |
| 12+ | 12·4 (99) | 12·6 (97) | 0·966 |
| 10-11 | 23·4 (187) | 23·5 (180) | 0·982 |
| 0-9 | 64·2 (512) | 63·9 (490) | 0·921 |
| **Smoking Status** |  |  |  |
| Never smoker | 35·4 (286) | 35·1 (272) | 0·941 |
| Ex-smoker | 59·0 (477) | 59·1 (459) | 0·975 |
| Current smoker | 5·7 (46) | 5·8 (45) | 0·984 |
| **Body mass index (kg/m²)** |  |  |  |
| <18.50 | 6·6 (47) | 6·6 (46) | 0·999 |
| 18.50-24.99 | 51·4 (368) | 51·1 (356) | 0·936 |
| 25.00-29.99 | 32·5 (233) | 33·1 (231) | 0·891 |
| ≥30.00 | 9·5 (68) | 9·2 (64) | 0·953 |
| **Cognitive function** |  |  |  |
| Normal (26-30) | 74·0 (582) | 74·3 (564) | 0·908 |
| Mildly impaired (22-25) | 15·0 (118) | 15·3 (116) | 0·949 |
| Moderately impaired (18-21) | 4·9 (39) | 5·0 (38) | 0·984 |
| Severely impaired (0-17) | 6·1 (48) | 5·4 (41) | 0·888 |
| **FI-CD (mean, SD)** | 0·22 (0.12) | 0·21 (0.12) | 0·605 |
|  |  |  |  |

^a^ The data are % (n) except where specified; denominators vary due to missing values.

^b^ p values are calculated from the test of proportions except for the last row where Student t-test was used for no significant differences between FI-CDs in the two samples.

Table S3. The area under the receiving operating characteristic (AUC) with 95% confidence intervals for different versions of the FI and Fried phenotype in the subsample of participants for whom the Fried phenotype was available (n=552).

|  | AUC | 95% CI |
| --- | --- | --- |
| **FI-B** | 0.61 | 0.57 – 0.66 |
| **FI-CD** | 0.64 | 0.60 – 0.69 |
| **FI-CD + FI-B** | 0.68 | 0.63 – 0.73 |
| **FI-CD + FI-B + sex** | 0.72 | 0.68 – 0.76 |
| **FI-CD + FI-B + sex + Fried phenotype** | 0.72 | 0.68 – 0.77 |
| **Fried phenotype** | 0.58 | 0.53 – 0.63 |
